# Isolation and identification of mycorrhizal helper bacteria of *Vaccinium uliginosum* and their interaction with mycorrhizal fungi

**DOI:** 10.3389/fmicb.2023.1180319

**Published:** 2023-04-18

**Authors:** Zhiyu Yang, Hui Dong, Sai Zhang, Jing Jiang, Haifeng Zhu, Hongyi Yang, Lili Li

**Affiliations:** ^1^Key Laboratory of Saline-alkali Vegetation Ecology Restoration (Northeast Forestry University), Ministry of Education, Harbin, China; ^2^College of Life Science, Northeast Forestry University, Harbin, China; ^3^Institute of Forestry Science of Heilongjiang Province, Harbin, China

**Keywords:** blueberry, ericoid mycorrhizal fungi, mycorrhizal helper bacteria, mycorrhiza, interactions

## Abstract

Mycorrhizal helper bacteria (MHB) can promote mycorrhizal fungal colonization and form mycorrhizal symbiosis structures. To investigate the effect of interactions between mycorrhizal beneficial microorganisms on the growth of blueberry, 45 strains of bacteria isolated from the rhizosphere soil of *Vaccinium uliginosum* were screened for potential MHB strains using the dry-plate confrontation assay and the bacterial extracellular metabolite promotion method. The results showed that the growth rate of mycelium of *Oidiodendron maius* 143, an ericoid mycorrhizal fungal strain, was increased by 33.33 and 77.77% for bacterial strains L6 and LM3, respectively, compared with the control in the dry-plate confrontation assay. In addition, the extracellular metabolites of L6 and LM3 significantly promoted the growth of *O. maius* 143 mycelium with an average growth rate of 40.9 and 57.1%, respectively, the cell wall-degrading enzyme activities and genes of *O. maius* 143 was significantly increased. Therefore, L6 and LM3 were preliminarily identified as potential MHB strains. In addition, the co-inoculated treatments significantly increased blueberry growth; increased the nitrate reductase, glutamate dehydrogenase, glutamine synthetase, and glutamate synthase activities in the leaves; and promoted nutrient uptake in blueberry. Based on the physiological, and 16S rDNA gene molecular analyses, we initially identified strain L6 as *Paenarthrobacter nicotinovorans* and LM3 as *Bacillus circulans*. Metabolomic analysis revealed that mycelial exudates contain large amounts of sugars, organic acids and amino acids, which can be used as substrates to stimulate the growth of MHB. In conclusion, L6 and LM3 and *O. maius* 143 promote each other’s growth, while co-inoculation of L6 and LM3 with *O. maius* 143 can promote the growth of blueberry seedlings, providing a theoretical basis for further studies on the mechanism of ericoid mycorrhizal fungi-MHB-blueberry interactions. It laid the technical foundation for the exploitation of biocontrol strain resources and the development of biological fertilizer.

## Introduction

Blueberry is a perennial shrub and small berry fruit tree, and it originated in North America (Wu et al., 2011; Chu et al., 2018). Its fruit is rich in anthocyanins and has low sugar and fat contents and high antioxidant capacity. It is listed by the International Food and Agriculture Organization as one of the top five health foods for human beings and is a new generation of fruit tree species with high economic value and broad construction prospects (Li et al., 2016; Wang et al., 2017; Lin et al., 2020). In recent years, blueberry has become representative of healthy berries; the worldwide demand is still rising, and the situation of a shortage of supply still exists (Rasmussen et al., 2005; Siddiq and Dolan, 2017). Therefore, the expansion of the blueberry planting area and improvement in blueberry production during the process of blueberry cultivation are urgently needed to solve the problem.

As blueberry has a fibrous root system without root hairs, it has a weak ability to absorb nutrients, and most ericoid plants grow in harsh and infertile environments (Wei et al., 2016a). To effectively obtain resources from the soil, blueberries have evolved a variety of adaptive strategies. In addition to regulating the morphological and physiological characteristics of their root systems, the plants can also form mycorrhizae with soil microorganisms represented by ericoid mycorrhizal fungi (ErMF) to promote the growth of the host plants, improve the utilization and uptake of elements by the plants, and thus promote fruit yield (Cairney and Meharg, 2003; Kosola et al., 2007). Many researchers have isolated and identified mycorrhizal fungi from *Vaccinium uliginosum* (Yang et al., 2018), *Rhododendron obtusum* var. *kaempferi* (Usuki et al., 2003) and *Calluna vulgaris* (Diaz et al., 2006). Currently, most of the artificially cultivable fungi that have been isolated from rhododendron mycorrhizal species belong to the *Ascomycetes*. Members of *Ascomycetes*, especially *Helotiales*, are the most widely reported ErMF. *Oidiodendron* of the subphylum rhododendron has also been frequently isolated. Tian et al. (2011) isolated *O. maius* from *Rhododendron grandiflorum* in Yunnan, China, and found that it accounted for 18.4% of the mycorrhizal fungi isolated. Wei et al. (2016b) isolated *O. maius* Om 19 from *R. fortunei* and found that the fresh and dry weights of *R. fortunei* 2 months after inoculation with Om 19 were greater than those of uninoculated plants, and the total nitrogen absorbed by plants inoculated with Om 19 was also higher than that of the uninoculated control. In recent years, with the development of proteomics and transcriptomics and their application in the study of mycorrhizal fungi, many scholars have used *O. maius* as a model species to study the molecular mechanisms of host promotion and resistance using proteomics and transcriptomics and have achieved great breakthroughs (Abbà et al., 2011; Khouja et al., 2013, 2014; Chiapello et al., 2015).

Recent studies have shown that mycorrhizal interactions are complex microecosystems, and mycorrhizal-plant symbioses have close interactions with rhizosphere fungi, bacteria, actinomycetes, and other microorganisms at all levels of physical structure, active ingredient metabolism, and functional performance. Some of these bacterial groups play an important role in promoting mycorrhizal synthesis and plant growth (Frey-Klett et al., 2005; Bonfante and Anca, 2009). These bacteria are called mycorrhizal helper bacteria (MHB). MHB can specifically bind to mycorrhizal fungi, promote mycorrhizal fungal spore germination and mycelial growth, increase the mycorrhizal infestation rate, promote mycorrhizal fungal colonization and growth in host plant roots, and thus indirectly promote plant growth (Garbaye, 1994; Frey-Klett and Garbaye, 2005). Heydari and Pirzad (2020) found that co-inoculation of mycorrhizal fungi with sulfur oxidizing bacteria (SOB) enhanced soil fertility, improved soil physicochemical properties, and increased soil enzyme activity, thereby promoting host plant growth. Kurth et al. (2015) used transcriptome sequencing to explore the interaction between *Quercus robur*-*Streptomyces* sp. AcH505 and *Piloderma croceum* (ectomycorrhizal fungi). Under *Streptomyces* induction, *Q. robur* root parts recognized xenobiotics, and root epidermal cell development genes and root defense genes were differentially expressed compared with the control. In addition, more differentially expressed genes were found in newly developed roots than in old roots, proving that root development was regulated by and more favorable to mycorrhizal recognition and infestation after *Streptomyces* AcH505 induction. Currently, most reports on MHB focus on arbuscular mycorrhizal fungi (AMF) and ectomycorrhizal fungi (Deveau et al., 2018; Sangwan and Prasanna, 2022); however, the mechanism of MHB involved in blueberry growth and ericoid species mycorrhizal formation is not clear.

In this study, we screened and identified MHB from the rhizosphere soil of wild *V. uliginosum* and investigated the effects of MHB on the two participants of symbiosis (host plant and ErMF) separately, including growth and factors related to mutual recognition during symbiosis. The synergistic effect of MHB and ErMF on mycorrhizal formation was further verified. At the same time, excellent strains of MHB that can effectively promote ERM formation in Ericaceae were obtained. Coinoculation of MHB with ErMF can promote the growth of blueberry seedlings. This will lay the foundation for the preparation of mycorrhizal mixes with mycorrhizal fungi and the development of blueberry biofertilizer, so as to increase growth and reduce the use of fertilizer in blueberry.

## Materials and methods

### Fungal isolates

The mycorrhizal fungi were obtained from *O. maius* 143, an ericaceous mycorrhizal fungus conserved in the Microbiology Laboratory of Northeastern Forestry University, which was isolated from blueberry roots.

### Isolation and purification of bacteria from *Vaccinium uliginosum* rhizosphere soil

The steps were as follows: Remove the humus from the roots of *V. uliginosum* and dig 10–20 cm along the root layer of the plant. Shake off the large particles of soil from the roots of *V. uliginosum*, collect the soil connected to the hairy roots of the plants, put the rhizosphere soil in a sterile sealed bag, and store it in a refrigerator at 4°C.

Weigh 1 g of soil sample, add 100 mL of physiological saline, incubate the mixture at 37°C, 180 r/min for 30 min with shaking, and then let it stand for 60 min. Aspirate 1 mL of supernatant, add this to 9 mL of sterile water in a test tube, dilute 10 times, and repeat to 10^−5^ and 10^−6^. Aspirate 50 μL the above solution, and apply it evenly to Tryptose Soya Agar (TSA: 15.0 g/L tryptone, 5.0 g/L soy peptone, 5.0 g/L NaCl, and 15.0 g/L agar) solid medium at each dilution for three parallel experiments. Put the plates in a 37°C incubator for 24 h and observe the colony morphology. Pick single colonies with different colony morphologies, transfer them to new solid TSA medium by the plate scribing method, incubate these at 37°C for 24 h, pick single colonies and transfer these to liquid TSA medium, incubate at 180 r/min, 37°C overnight, remove the bacterial solution and continue to coat this onto TSA medium, repeat three times for purification, and finally obtain a single strain with consistent colony characteristics (Zhao et al., 2014).

### Screening of MHB

The isolated bacteria were inoculated in 100 mL of TSA liquid medium and incubated at 37°C and 180 r/min for 24 h. The flat dishes of strain *O. maius* 143 were incubated for 7 d and punched with a sterile punch, and the fungal blocks were placed on a petri dish with a layer of 15% agar at the bottom. There were three blocks per dish, placed in a triangular shape, and 30 μL of bacterial suspension was aspirated and added dropwise to each fungal block repeatedly to ensure that the bacterial suspension was placed evenly on the surface of the fungus. The fungal blocks with liquid TSA medium as the control were incubated in the dark at 25°C, and 0.01 mol/L glucose solution was added dropwise every 3 d to prevent the fungus from drying out. After 7 d of fungal growth, the mycelial growth was observed and recorded (Dunstan et al., 1998).

### Growth, enzyme activities, and real-time PCR assay of *Oidiodendron maius* 143

Two strains of potential MHB, L6 and LM3, were inoculated with 100 mL of Luria-Bertani (LB) medium and incubated at 180 r/min for 24 h. The broth was filtered at 4°C for 15 min at 10,000 r/min. *O. maius* 143 was grown on potato dextrose agar (PDA) medium for 14 d at 28°C. The fermentation broth product was filtered after centrifugation at 10,000 r/min for 15 min. After 14 d of growth at 28°C, the slices were removed with a sterile punch and added to triangular flasks containing 100 mL of PDA liquid medium with four blocks and 2 mL of fermentation solution product per flask. Then 2 mL of LB medium was added to the control group and grown at 28°C 150 r/min for 10 d. The mycelium was dried at 80°C for 24 h and then weighed.

The mycelium was collected and rinsed with sterile water to remove the medium, and the mycelium bodies were ground and resuspended in 1× PBS solution. The supernatant of the solution was ultrasonically decomposed and used to determine the cell wall-degrading enzyme activity. Chitinase, β-1,4-glucanase, polygalacturonase, and β-glucosidase activities in the reaction solution were determined by the DNS colorimetric method using 1% colloidal chitin solution, 1% sodium carboxymethylcellulose, 1% polygalacturonase solution, and 5% salicin solution as substrates. The amount of enzyme required to catalyze the release of 1 μmol of reducing sugar per minute of substrate was taken as one unit of enzyme activity (Mucha et al., 2006; Tang et al., 2008; Meng et al., 2021).

The hyphae were collected and subjected to total RNA extraction using a Fungal Total RNA Isolation Kit (Sangon Biotech, Shanghai, China) according to the manufacturer’s instructions. Approximately 1 μg of the total RNA was reverse-transcribed into cDNA using a HiFiScript cDNA Synthesis Kit (CW2569, Cwbio, China). Homologous genes in the *O. maius* 143 genome (GCA_000827325.1), which were reported in *L. bicolor* and differentially expressed during mycorrhizal formation or interaction with MHB (Deveau et al., 2007; Wang et al., 2022), were queried in BLAST, and the Primer-Blast program of the NCBI was used to design quantitative PCR primers (Supplementary Table S1). The reaction mixture consisted of 2 μL of template cDNA, 10 μL of ChamQ™ SYBR qPCR Master Mix (Q311–02, Vazyme, China), 0.4 μL each of the forward and reverse primers (10 mM), and 7.2 μL of RNA-free water. Amplification was performed with a StepOne Thermal Cycler (Applied Biosystems 7500, USA), which consisted of 40 cycles of denaturation at 95°C for 30 s, annealing at 95°C for 5 s, and extension at 50°C for 30 s. The results were analyzed using the 2 ^–△△^CT method.

### Collection and determination of *Oidiodendron maius* 143 mycelial exudates

In this experiment, mycelial exudates were collected by culturing *O. maius* 143 in two partitioned petri dishes (90 × 15 mm): 25 mL of solid M medium was added to the left compartment and 4 mL of solid M medium (without sucrose, EDTA and vitamins) was added to the right side, near the horizontal partition, to form a slant (Filion et al., 1999). A 9 mm diameter block of *O. maius* 143 was placed on the left side of the culture compartment. Sealed petri dishes were incubated at 27°C under dark conditions. After eight weeks, when the fungal hyphae started to grow across the transverse septum on the slant, 10 mL of liquid M medium (without sucrose and vitamins) was added to the right compartment. After four weeks, when most of the liquid medium surface in the right culture chamber was covered by *O. maius* 143 fungal mycelia, the medium was harvested, with three replicates per group (Bharadwaj et al., 2012). The medium was filtered through a 0.22 μm membrane, and 1,500 μL was taken into a 2 mL centrifuge tube, concentrated and dried under vacuum, 500 μL of methanol was added, shaken for 60 s, mixed thoroughly, and centrifuged at 12,000 rpm for 10 min at 4°C. 450 μL of supernatant was removed and transferred to a new 2 mL centrifuge tube, concentrated and dried under vacuum; 150 μL of 2-chlorophenylalanine (4 ppm) 80% methanol solution was added. The supernatant was filtered through a 0.22 μm membrane to obtain the sample to be tested; the remaining sample to be tested was subjected to liquid chromatography-mass spectrometry (LC–MS).

### Plant material and treatment

The two potential strains of MHB and *O. maius* 143 were activated in advance in LB and PDA medium, respectively. Vermiculite and charcoal soil were mixed evenly in a ratio of 1:3, added with appropriate distilled water to adjust the soil humidity, and added to a 400 mL flowerpot; one-third of the soil was added to each flowerpot. Autoclaving (121°C) was conducted for 90 min, and blueberry seedlings with similar growth status and plant height were selected and inserted into the soil. The experiment mainly consisted of the following treatments: (1) single inoculation of *O. maius* 143: after 2 weeks of PDA culture, three fungal blocks were punched with a hole punch and inoculated into the soil 1 cm deep from the roots of sterile blueberry seedlings, taking care that the fungus did not touch the blueberry seedlings during the operation. (2) Single inoculation of L6: 2 mL of bacterial suspension (10^7^ colony forming units (cfu)/g soil) was poured along the edge of the L6 bacterial culture inoculum that was grown in 50 mL LB medium at 37°C, with 180 r/min shaking for 24 h. (3) Single inoculation of LM3: 2 mL of bacterial suspension (10^7^ cfu/g soil) was slowly poured into the Histoplasma vial along the edge. LM3 bacterial culture inoculum was grown in 50 mL LB medium at 37°C, with 180 r/min shaking for 24 h. (4) Co-inoculation 143 + L6: after 2 weeks of PDA culture, three fungal blocks were punched with a sterile punch and inoculated in soil 1 cm deep from the roots of sterile blueberry seedlings, and 2 mL of bacterial suspension (10^7^ cfu/g soil) was slowly poured into the flowerpots along the edge. L6 bacterial culture inoculum was grown in 50 mL LB medium at 37°C and shaken at 180 r/min for 24 h. (5) Co-inoculation 143 + LM3: after 2 weeks of PDA culture, three fungal blocks were punched with a sterile punch and inoculated in soil 1 cm deep from the roots of sterile blueberry seedlings, and 2 mL of bacterial suspension (10^7^ cfu/g soil) was poured slowly along the edge into the flowerpots. LM3 bacterial culture inoculum was grown in 50 mL LB medium at 37°C with 180 r/min shaking for 24 h. (6) Control group: no microbial inoculation treatment; the remaining conditions were consistent with those of the experimental group. All seedlings were grown in a greenhouse at 28°C (12 h light, 12 h dark) for 2 months.

### Measurement of blueberry growth

Plant materials were collected after 2 months, and the plant height and root length were measured with a ruler; plant growth was measured with a balance; and root activity was determined using a modified 2,3,5-triphenyltetrazolium chloride method (Peng et al., 2022). For each treatment group, 1.0 g of fresh leaves was placed in a mortar, ground with a small amount of quartz sand, and then extracted with 20 mL of 80% acetone and centrifuged at 10,000 r/min for 10 min at 4°C. The absorbance values of the supernatant were measured at 645 nm and 663 nm, and the chlorophyll content was determined. The plant roots were stained with the Tryptan blue staining method (Liu et al., 2016). The roots of blueberry seedlings were carefully removed and washed, and the water was blotted with absorbent paper. The mycorrhizal infestation rate is calculated as follows: (number of mycorrhizal root segments formed/total number of root segments) × 100%.

Fungal colonization was detected by qRT-PCR as follows: The *O. maius* 143 genomic DNA was isolated from the mycelia using the modified cetyltrimethylammonium bromide method (Gardes and Bruns, 1993). The internal transcribed spacer (ITS) region was amplified using the G143F (TGAGACCAAAGTCCCCTTCAACCAAAA) and G143R (TTGGTGGCAATCAAAAGAGATAC) primers. The tubes were incubated at 94°C for 2 min and then subjected to 30 cycles as follows: 94°C for 30 s, 60°C for 30 s, and 72°C for 30 s; a final incubation was carried out for another 5 min at 72°C. The PCR product was purified by a Gel Extraction Kit (Sangon Biotech, Shanghai, China) and then transligated with the pMD18-T vector. Plasmids were extracted using a Plasmid Mini Kit (Omega Bio-tek, Norcross, GA, USA). The plasmid DNA was diluted in a gradient, the DNA concentration (ng/μL) was measured by a Nanodrop, and the copy number of the corresponding concentration was calculated according to the following formula:

NC=(K×Na)/660×L,

where K is the sample concentration, Na is Avogadro’s constant 6.02 × 10^23^, and L is the sum of the fungal DNA fragment size and the plasmid fragment size.

Fluorescent quantitative PCR was performed on the diluted plasmid DNA. Three replicates of each sample were made, and the standard curve was made with the corresponding copy number as the *X*-axis and the obtained Cq value as the *Y*-axis.

The DNA of blueberry roots was extracted using an AxyPrep Multisource Genomic DNA Miniprep Kit (Axygen Biosciences, CA, USA), and the DNA of blueberry roots was used as the experimental material for fluorescent quantitative PCRs.

### Leaf enzyme activities

The activities of nitrate reductase (NR), glutamate dehydrogenase (GDH), glutamine synthetase (GS), and glutamate synthase (GOGAT) were determined spectrophotometrically as described below (Seith et al., 1994; Lin and Kao, 1996) with some modifications.

For the determination of NR viability, 1.0 g of fresh leaves was weighed into a pre-cooled mortar, a small amount of silica was added, the leaves were ground into a homogenate in liquid nitrogen and were centrifuged at 12,000 r/min for 10 min, and the supernatant was used as the crude enzyme. Then, 1 mL of 30% trichloroacetic acid was added to a blank tube, followed by the addition of 0.2 mL of the above crude enzyme solution, 0.1 mol/L KNO_3_ 0.5 mL and 2 mg/mL NADH solution 0.3 mL; the tubes were kept warm for 30 min at 25°C. The reaction was terminated by adding 1 mL of 30% trichloroacetic acid to the three replicate tubes followed by adequate shaking; 2 mL of sulfonamide reagent and 2 mL of α-naphthylamine reagent were added to each of the four tubes, shaken well, and left for 15 min. The absorbance of the supernatant was measured at 520 nm, and the amount of NO^2−^ μg produced per gram of fresh weight per hour was expressed as the unit of enzyme activity (μg/g/h).

For the determination of GDH viability, the crude enzyme was prepared according to the NR enzyme activity determination. The reaction solution was 2.8 mL (containing 60 mmol/L L-glutamic acid, 1.6 mmol/L with NAD^+^, and 360 mmol/L Tris–HCl buffer, pH 8.2); 0.2 mL of the crude enzyme extract was added and shaken well; the absorbance was recorded at 340 nm at 1 min intervals; and the reaction solution without glutamic acid was used as the control. A change in light absorption of 0.001 per 1 min in the reaction system was the unit of enzyme activity (nmol Glu/min/g).

For the determination of GS viability, 1.0 g of fresh leaves was weighed and ground in 2 mL of extraction solution (25 mmol/L Tris–HCl (pH = 7.6), 1 mmol/L MgCl_2_, 1 mmol/L EDTA, 14 mmol/L mercaptoethanol, and 1 g PVP) in liquid nitrogen, which was then centrifuged at 12,000 r/min for 10 min. Then, 0.1 mL of the supernatant was added to 0.3 mL of imidazole hydrochloride buffer (250 mmol/L, pH 7.0), 0.2 mL of sodium glutamate (300 mmol/L), 0.2 mL of ATP-Na2 (30 mmol/L), and 0.1 mL of MgSO_4_ (500 mmol/L); vortexed for 10 s; and shaken for 10 min, followed by placing in a 25°C water bath and shaking for 10 min, after which 0.1 mL hydroxylamine was added, placed in a 25°C water bath, and shaken for 20 min. The final step was the addition of the termination solution (0.37 M FeCl_3_ 0.4 mL) to the centrifuge tube to terminate the reaction. The absorbance of the supernatant was measured at 520 nm, and the GS activity (μmol/g/h) was calculated as the amount of γ-glutamyl hydroxamic acid produced per unit weight per unit time.

For the determination of GOGAT activity, 1.0 g of fresh leaves was added to 4 mL of pre-chilled 0.05 mol/L Tris–HCl buffer solution (pH 7.6) and ground in an ice bath. The mixture was centrifuged (12,000 r/min) for 10 min, and the supernatant was used as the crude enzyme. Then, 20 mmol/L L-glutamine 0.4 mL, 20 mmol/L α-ketoglutarate 0.5 mL, 10 mmol/L KCl 0.1 mL, 3 mmol/L NADH (nicotinamide adenine dinucleotide) 0.2 mL and 0.3 mL of enzyme solution were added to the cuvette. The absorbance value of the supernatant was measured at 340 nm, and GOGAT activity was calculated as the decrease in NADH per unit weight per unit time (μmol/g/h).

### Identification of potential MHB strains

Phenotypic tests were performed for the preliminary identification of MHB strains. Isolates were cultured on TSA at 28°C for 2–3 d. Colony morphology was determined, and morphology of cells was determined using a compound microscopy at a magnification of × 1,000. For 16S rDNA gene sequence analysis: inoculate the screened MHB into seed medium, incubate at 37°C, 180 r/min with vibration for 48 h, centrifuge at 12,000 r/min for 5 min to collect the bacterium, and extract the bacterial genomic DNA using the Genomic DNA Extraction Kit (Tiangen Biochemistry). The 16S rDNA gene universal primers 27F (5′-AGAGTTTGATCCTGGCTCAG-3′) and 1492R (5′-GGTTACCTTGTTACGACTT-3′)were used for PCR amplification of genomic DNA; the reaction system was as follows: 2× Taq PCR Mastermix 25 μL, 10 mmol/L 27F primer 1 μL, 10 mmol/L 1492R primer 1 μL, DNA PCR conditions: 94°C for 3 min; 94°C for 30 s, 55°C for 30 s, 72°C for 1 min, 30 cycles; 72°C for 5 min, stored at 4°C. PCR products were detected by 1% agarose gel electrophoresis and purified using a Universal DNA Purification Recovery Kit (Tiangen Biochemistry). The sequencing results were compared with BlastN on NCBI, and the sequences were submitted to GenBank to obtain the gene accession numbers. The 16S rDNA gene sequences of strains with high similarity rates were used to construct phylogenetic trees with MEGA X software for strain taxonomic status identification (Sambrook et al., 2001).

### Growth promotion and stress tolerance analysis

Nitrogen fixation activity: L6 and LM3 pure bacterial isolates were inoculated on solid nitrogen-free medium, incubated at 37°C for 3 d, and transferred three times consecutively; the bacteria that grew stably were considered to be able to fix atmospheric nitrogen (Kaleh et al., 2022).

Phosphate solubilization activity: L6 and LM3 pure bacterial isolates were collected by centrifugation at 12,000 r/min for 5 min and diluted with sterile water to OD600 nm = 0.6. Then, 1 mL of bacterial solution was inoculated into 100 mL of inorganic phosphorus medium, and LB was added in an equal amount as the control. Soluble P was evaluated from the standard curve of KH_2_PO_4_. The absorbance value of the supernatant was measured at 882 nm after a 30 min reaction (Pande et al., 2017).

Indole-acetic-acid (IAA) production: L-tryptophan filtered through a 0.22 μm membrane was added to 100 mL LB medium to yield a final concentration of 100 mg/L. The pure bacterial isolates were incubated at 25°C and 180 r/min for 48 h and were then centrifuged at 4°C and 12,000 r/min for 20 min. Then, 2 mL of the clear solution was added to an equal volume of Salkowski’s color development solution in the dark and left to stand for 30 min away from light. The standard curve was prepared. After 30 min color development, the absorbance value of the supernatant was measured at 530 nm (Yousef, 2018).

1-Aminocyclopropane-1-carboxylic acid (ACC) deaminase activity: the quantitative estimation of the ACC deaminase activity of bacterial isolates was measured by estimating α-ketobutyrate and ammonia produced from the cleavage of ACC by the enzyme ACC deaminase following the standard protocol of Penrose and Glick (2003). The estimation of α-ketobutyrate was determined in each sample by comparing it with a standard curve of α-ketobutyrate.

Siderophore production: The strains were spotted on chrome-azurol S (CAS) medium and incubated at 28°C for 7 d. After the colonies had grown, the formation of orange iron carrier secretion circles was observed (Schwyn and Neilands, 1987).

Pathogenic bacterial antagonistic ability assay: the plate standoff method was used. L6 and LM3 were incubated at 37°C and 180 r/min for 24 h to make a bacterial suspension, and the pathogenic bacterial cake was placed in the center of solid PDA with a sterile punch. Then, 5 μL of bacterial suspension was added dropwise around the cake, with no inoculation of bacteria as the control, and incubated in an inverted incubator at 25°C. The growth of pathogenic bacteria was recorded and observed daily to calculate the inhibition rate: Inhibition rate (%) = (control colony diameter − treated colony diameter)/ (control colony diameter) × 100. Heavy metal antagonism assay: L6 and LM3 were incubated overnight at 37°C at 180 r/min. The bacteria were collected by centrifugation at 12,000 r/min for 5 min and diluted in sterile water to OD_600_ nm = 0.6. Then, 100 μL of bacterial solution was removed and added to 5 mL of LB medium containing 0.7% agar at about 50°C, mixed well, and poured on the solid LB medium that had solidified. Sterilized filter paper sheets were placed in the center of the plate, and 10 μL of 50 mmol/L Cd^2+^, 100 mmol/L Zn^2+^, and 400 mmol/L Cu^2+^ were added dropwise to the filter paper sheets and incubated in an incubator at 37°C for 48 h. The diameter of the transparent circle was recorded using *Bacillus subtilis* as the control.

### Data analysis

Five treatments were set up for each trial. All reported data were statistically analyzed using one-way ANOVA (one-way analysis of variance) and Tukey’s significant difference (HSD) *post hoc* test. Significant differences were based on Tukey’s HSD *p* < 0.05. Statistical analysis was performed using SPSS 26.0 software. Metabolic data were analyzed by Suzhou BioNovoGene Biomedical Technology Co., Ltd., China.

## Results

### Promotional effect of MHB potential strains on *Oidiodendron maius* 143

**Figure 1:**
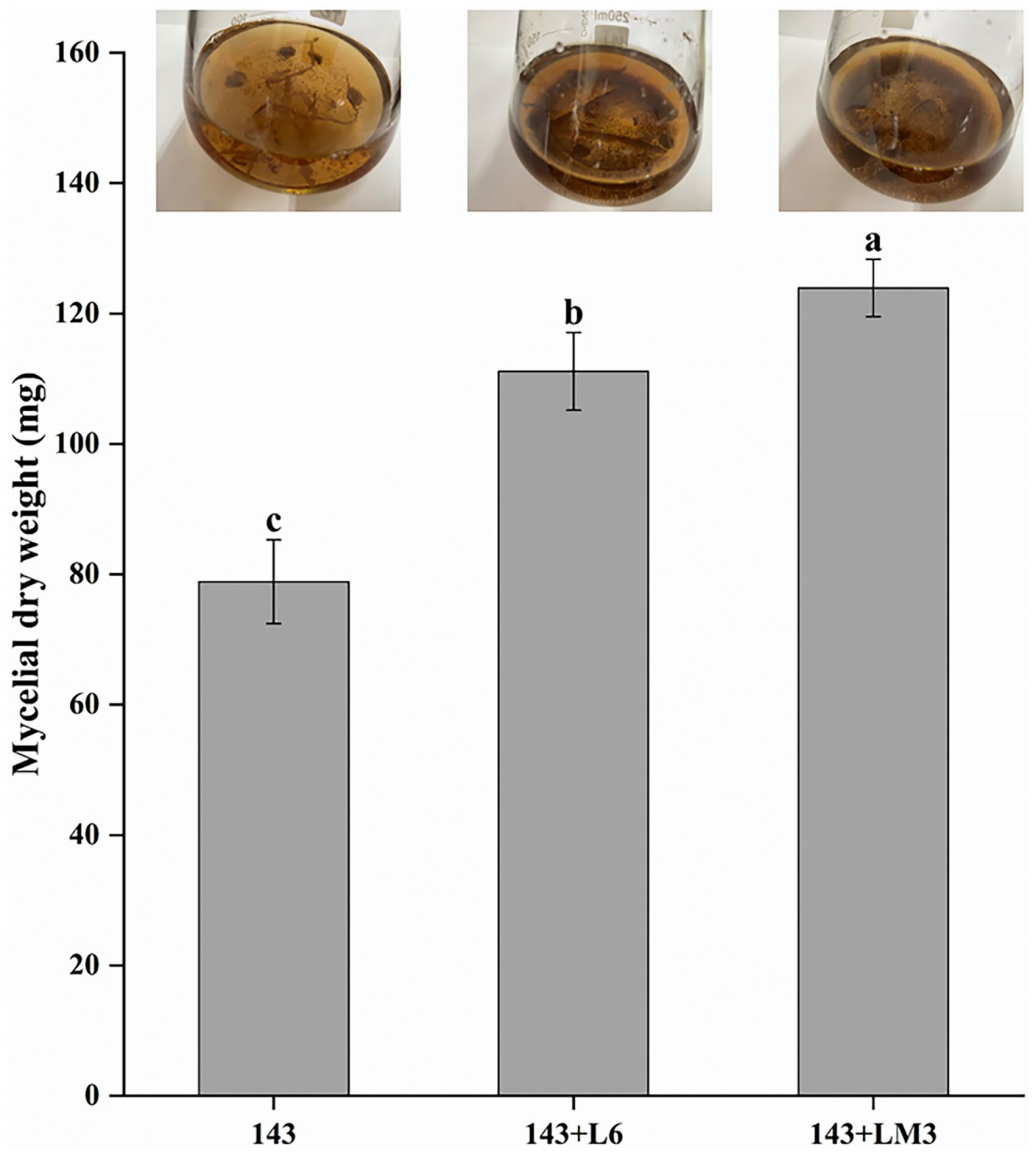
Effects of the fermented product of L6 or LM3 on *Oidiodendron maius* 143 growth. Mycelial dry weight of 143 in YPD liquid media supplemented with the fermentation broth of L6 or the fermentation broth of LM3. 143 is *O. maius* 143; 143 + L6 is the co-inoculation of the *O. maius* 143 and L6 isolates; and 143 + LM3 is the co-inoculation of the *O. maius* 143 and LM3 isolates. The treatments labeled with different letters are significantly different according to Tukey’s HSD at *p <* 0.05. Bars represent the standard deviations of the means.

Rhizosphere bacteria were isolated and purified from the rhizosphere soil of *V. uliginosum*. A total of 45 strains of rhizosphere bacteria were classified by colony morphology and characteristics. The 45 isolated rhizobacteria were re-screened to obtain bacteria with mycorrhizal promotion potential. The 45 strains were tested against *O. maius* 143 in dry dishes, and it was found that after 3 d of dropping the bacterial suspension, the fungal mass was surrounded by translucent hyphae extending around; the 143 + L6 and 143 + LM3 hyphal length increased by 33.33 and 77.77%, respectively, compared with the control group. Strain L6 and LM3 can be tentatively considered to be strains with mycorrhizal helper bacterial potential. The co-cultivation of the L6 and LM3 extracellular metabolites with *O. maius* 143 showed that the L6 and LM3 extracellular metabolites also promoted the biomass of *O. maius* 143 to different degrees, and the dry weight of mycelium increased by 40.9 and 57.1%, respectively, compared with the control (Figure 1).

### The fermented product of potential MHB strains affected the enzyme activities and expression of genes related to the mycorrhizal formation of *Oidiodendron maius* 143 *in vitro*

**Figure 2:**
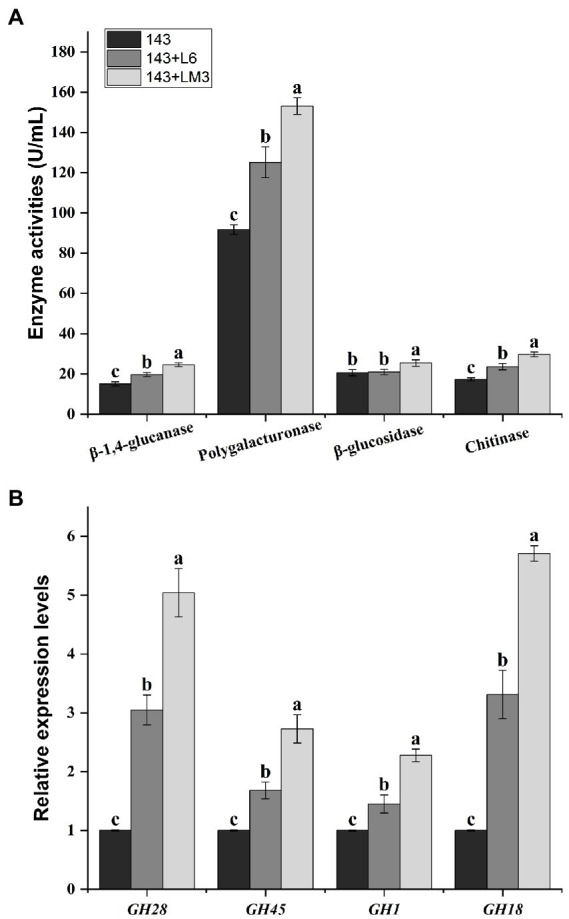
Effects of the fermented product of L6 or LM3 on the enzyme activities and gene expression of *O. maius* 143. **(A)** Effects of the fermented product of L6 or LM3 on the enzyme activities of *O. maius* 143. **(B)** Effects of L6 or LM3 on the expression of the mycorrhizal formation–related genes of *O. maius* 143. Treatments labeled with different letters are significantly different according to Tukey’s HSD at *p <* 0.05. Bars represent the standard deviations of the means.

Mycorrhizal fungi, when forming mycorrhizae with plants, secrete a large number of cell wall-degrading enzymes to disrupt plant cell walls and facilitate mycelial invasion to form symbioses. They mainly include chitinase, β-1,4-glucanase, β-glucosidase, and polygalacturonase, which play important roles in symbiosis. The results showed that the addition of L6 as well as LM3 to the supernatant of the fermentation broth significantly increased *O. maius* 143 chitinase, β-1,4-glucanase, and polygalacturonase compared to the control. The addition of the fermentation broth supernatant of LM3 increased *O. maius* 143 β-glucosidase by 22.9% compared to the control. In contrast, there was no significant difference in the β-glucosidase of *O. maius* 143 with the addition of the fermentation broth supernatant of L6 (Figure 2A).

The expression levels of chitinase (*GH18*), β-1,4-glucanase (*GH45*), β-glucosidase (*GH1*), and polygalacturonase (*GH28*) genes in mycelia were significantly increased in the three groups after adding bacterial fermentation broth, and there were significant differences among the different treatments (Figure 2B).

### Determination of *Oidiodendron maius* 143 mycelial exudates

The main components of *O. maius* 143 mycelial exudates were determined using LC–MS (Supplementary Table S2). Among them were 135 metabolites of sugars (fructose, arabinose and sucrose), organic acids and amino acids. The current study on mycelial exudate mainly focused on sugars, and this study found that both L6 and LM3 could grow well when fructose, arabinose and sucrose were the only carbon sources, and it was speculated that *O. maius* 143 has a symbiotic relationship with L6 and LM3.

### Potential MHB strain intercrossed with 143 to promote blueberry growth

The growth of blueberry seedlings was observed after 2 months in response to single inoculation with bacteria or mycorrhizal fungi and double inoculation with 143 + L6 and 143 + LM3 in the root zone of the blueberry seedlings. Both the single (bacterial or mycorrhizal fungal) and double inoculation treatments significantly promoted the growth of the blueberry seedlings, and the differences between the treatments were significant (Table 1). The growth of the blueberry seedlings with double inoculation 143 + L6 and 143 + LM3 was significantly better than that of the control (*p* < 0.05), with increases of 124 and 141%, respectively. Similarly, the plant biomass and root activity of the double inoculation treatment were twice as high as those of the control and were accompanied by increased chlorophyll II, which promoted photosynthesis in blueberry.

**Table 1:** Effect of the ericoid mycorrhizal fungi *Oidiodendron maius* 143 coinoculated with L6 or LM3 isolates on the plant growth and colonization rate.

Treatments	Shoot length (cm)	Root length (cm)	Biomass (g)	Root activity (μg/g/h)	Chlorophyl II (mg/g·FW)	Colonization rate (%)	Log (DNA copy numbers)
CK	8.87 ± 0.72f	0.79 ± 0.05d	0.52 ± 0.07d	80.56 ± 9.83e	1.23 ± 0.09e	0	0
143	17.64 ± 0.90c	1.21 ± 0.08c	1.04 ± 0.09b	116.99 ± 7.54c	1.78 ± 0.08c	31.8 ± 2.28c	5.21 ± 0.03c
L6	11.63 ± 1.15e	0.94 ± 0.14d	0.75 ± 0.07c	97.89 ± 8.16d	1.41 ± 0.10d	0	0
143 + L6	19.89 ± 1.11b	1.47 ± 0.10b	1.06 ± 0.11b	129.87 ± 9.88b	2.21 ± 0.13b	42 ± 3.67b	5.74 ± 0.02b
LM3	15.15 ± 0.87d	0.89 ± 0.09d	0.62 ± 0.11 dc	93.59 ± 4.29d	1.54 ± 0.09d	0	0
143 + LM3	21.41 ± 0.82a	1.81 ± 0.11a	1.36 ± 0.13a	153.80 ± 7.68a	2.55 ± 0.14a	48.4 ± 3.05a	6.75 ± 0.11a

The treatments labeled with the different letters are significantly different according to Tukey’ s HSD at *p* < 0.05. CK is control without any inoculums; 143 is *O. maius* 143; L6 is L6 isolate; 143 + L6 is *O. maius* 143 and L6 isolate; LM3 is LM3 isolate; 143 + LM3 is *O. maius* 143 and LM3 isolate.

**Figure 3:**
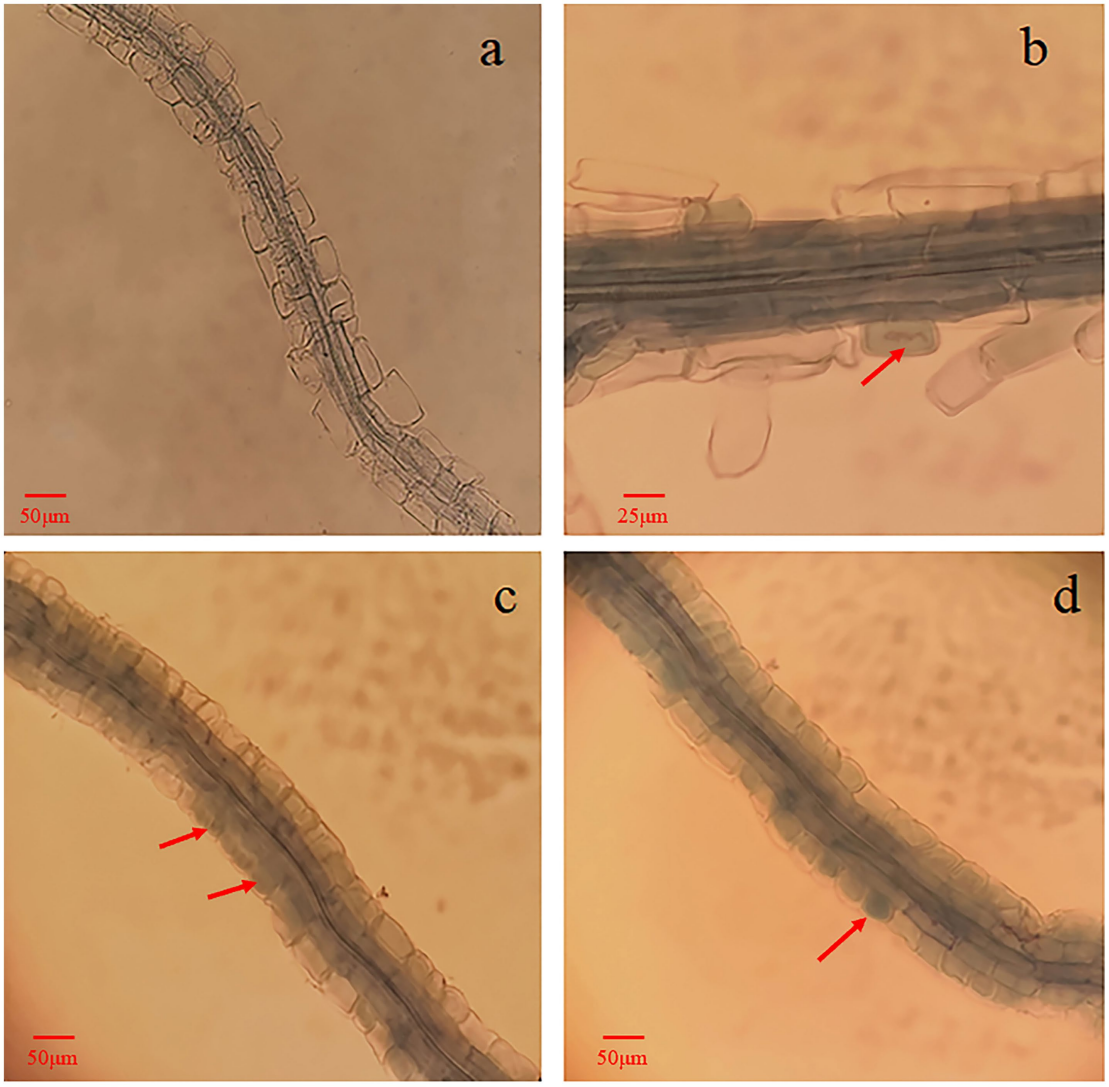
Microscopic observation of blueberry roots inoculated with *O. maius* 143. **(A)** Blueberry alone, roots of control seedlings (without inoculation) with no mycorrhizal colonization. **(B)** Blueberry with the mycorrhizal fungus *O. maius* 143. **(C)** Blueberry with the mycorrhizal fungus *O. maius* 143 and L6 isolates. **(D)** Blueberry with the mycorrhizal fungus *O. maius* 143 and LM3 isolate. Hyphal growth inside the epidermal cells of roots from inoculated plants is shown in **B**, **C**, and **D**. The red arrows are mycelial coils.

It was confirmed by the microscopic observation that fungal mycelia from the single inoculation with 143 as well as the double inoculation with 143 + L6 and 143 + LM3 colonized the blueberry roots. Upon Tryptan blue staining, mycelial nodules within the epidermal or cortical cells of the hairy roots filled the entire cell, and it was observed that the cells of some root segments were fully infested, forming typical intracellular mycelial nodules or mycelial circles (Figure 3). These characteristics were the same as those previously described for ErMF. The roots of uninoculated and singly inoculated blueberry seedlings with bacteria were clean and transparent, and no mycelia were observed. The mycelial colonization rates increased by 32 and 52% for co-inoculation with 143 + L6 and 143 + LM3, respectively, compared to 143 alone. The DNA copy numbers were calculated by qRT-PCR, and the Cq values obtained were inserted into the standard curve. We found that both strains of bacteria in the co-inoculation group were effective in increasing fungal colonization compared to the single inoculated strain 143, with the highest mycorrhizal fungal colonization in the LM3 and fungal co-inoculation treatment.

### Leaf enzyme activities

**Figure 4:**
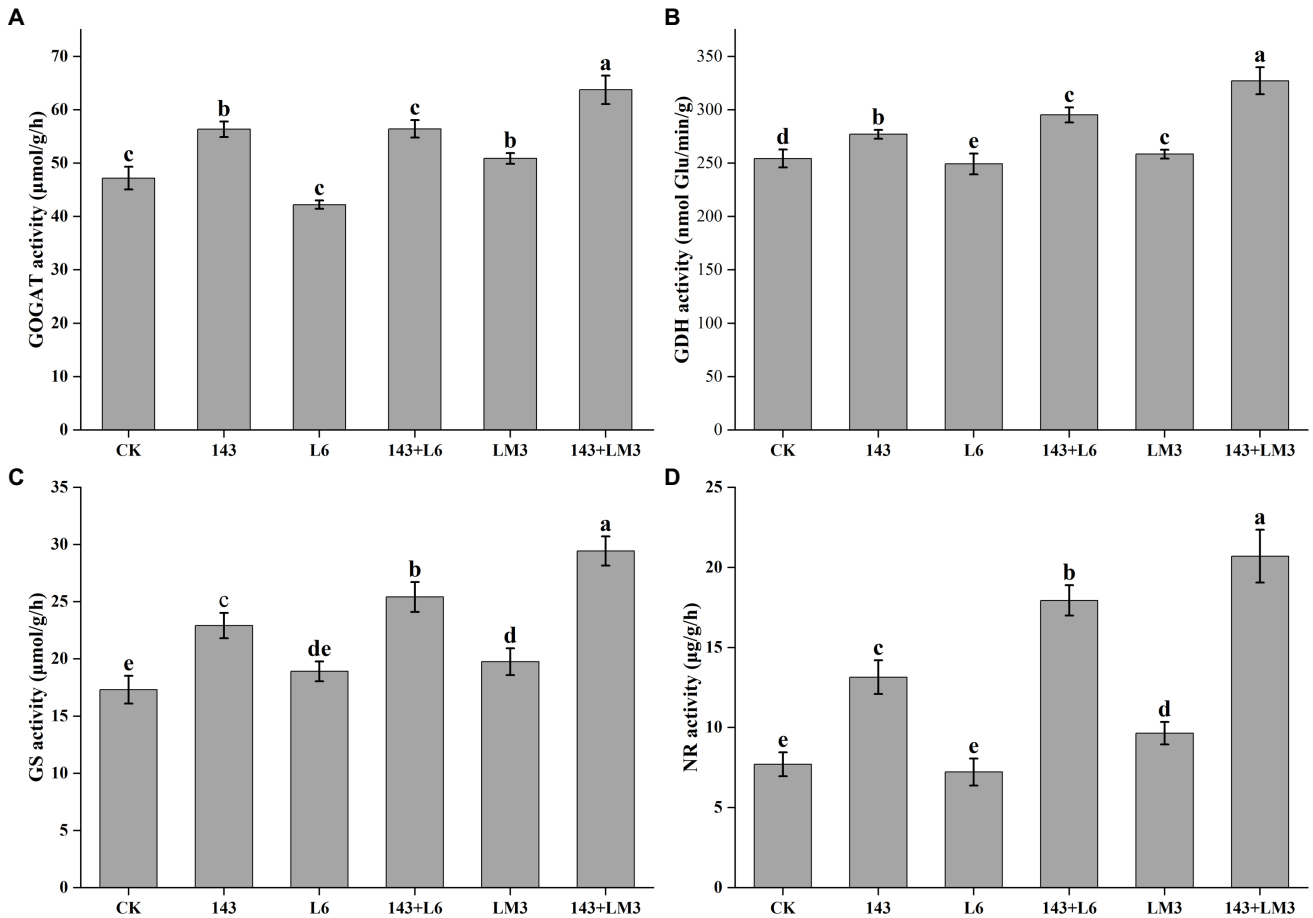
Effects of potential MHB strains and *O. maius* 143 on the enzyme activities of blueberry leaves. **(A)** Effect of potential MHB strains and *O. maius* 143 on the GOGAT activity of blueberry. **(B)** Effect of potential MHB strains and *O. maius* 143 on the GDH dismutase activity of blueberry. **(C)** Effect of potential MHB strains and *O. maius* 143 on the GS activity of blueberry. **(D)** Effect of potential MHB strains and *O. maius* 143 on the NR activity of blueberry. CK is the control without any inoculums. The treatments labeled with different letters are significantly different according to Tukey’s HSD at *p <* 0.05. Bars represent the standard deviations of the means.

The co-inoculated microbial treatment groups significantly increased the GOGAT, GDH, GS, and NR enzyme activities in the blueberry leaves, with significant differences among treatments. The GOGAT, GDH, GS, and NR enzyme activities increased by 19.6, 16, 46.7, and 132.8%, respectively, in the 143 + L6 treatment group compared to the control group; the L6 group slightly inhibited the GOGAT, GDH, and GS enzyme activities, while all other microbial treatment groups promoted the activities of all four enzymes. The GOGAT, GDH, GS, and NR enzyme activities increased by 35, 28.7, 70, and 168.8%, respectively, in the 143 + LM3 treatment group compared to the control group. Co-inoculation with 143 + LM3 resulted in higher sensitivity to the enzyme activity of blueberry leaves (Figure 4).

### Morphological and molecular biological identification of potential MHB strains

The two strains of bacteria grew well on the TSA medium; the L6 colonies were creamy white in the early stage and yellow in the later stage, with elevated colonies and a moist surface. The colonies of strain LM3 were slightly white and translucent, with neat edges and raised and slimy colonies.

**Figure 5:**
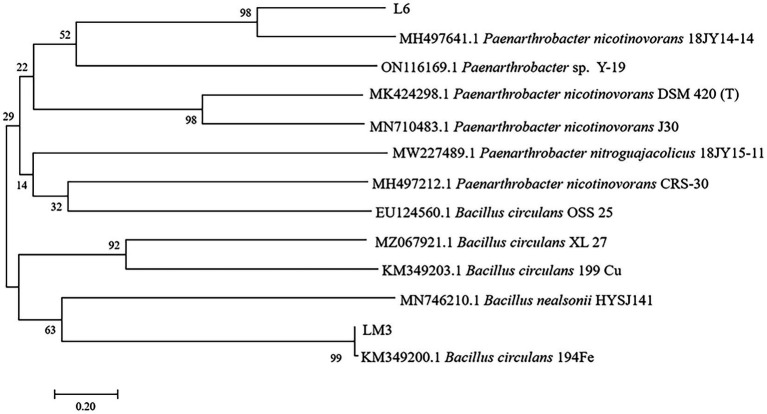
Neighbor-joining phylogenetic tree based on the 16S rDNA sequence data of potential MHB strains L6 and LM3 isolated from *Vaccinium uliginosum* rhizosphere soil. Numerical values above the branches indicate the bootstrap percentiles from 1,000 replicates. Bootstrap numbers higher than 50% are indicated. Horizontal branch lengths are proportional to the scale of the substitutions.

The 16S rDNA sequences of L6 and LM3 were sequenced and compared with the 16S rDNA sequences in GenBank, which showed that L6 was similar to *Paenarthrobacter nicotinovorans* (MH497641.1), with 99.46% similarity, and LM3 was similar to *Bacillus circulans* (KM349200.1), with 99.54% similarity. Based on the morphological and physicochemical characteristics of the strains and combined with 16S rDNA sequence analysis, strain L6 was tentatively identified as *P. nicotinovorans* and LM3 was tentatively identified as *B. circulans* (Figure 5).

### Analysis of L6 and LM3 for growth promotion and stress tolerance

To further explore the ecological functions of the MHB strains, L6 and LM3 were analyzed for their ability to promote growth and resist stress. The results (Table 2) showed that both strains had the following abilities: nitrogen fixation, phosphate solubilization, IAA production, and iron carrier production. L6 had better phosphate solubilization and iron carrier production than LM3, LM3 had twice the concentration of IAA compared with L6, and LM3 produced ACC deaminase with an enzyme activity of 3.75 U/mg, while L6 did not produce ACC deaminase. The resistance results (Table 3) showed that LM3 inhibited the growth of *F. sporotrichioides* by 51.6%, and L6 inhibited the growth of *F. sporotrichioides* and *B. dothidea*, with 31.25 and 38.6% inhibition rates, respectively. The antagonistic ability of L6 and LM3 against heavy metals Cd^2+^, Cu^2+^ and Zn^2+^ was investigated by the plate spot lawn standoff method with *B. subtilis* as the control. The results showed that the two strains of bacteria were significantly resistant to 50 mmol/L Cd^2+^, 400 mmol/L Cu^2+^, and 100 mmol/L Zn^2+^ based on measurements of the hyaline circle diameter, and LM3 was more resistant.

**Table 2:** Analysis of growth promoting ability of L6 and LM3.

MHB	Nitrogen fixation	Phosphorite-dissolving		IAA production capacity		ACC deaminase production capacity		Iron producing carrier color
	Qualitative screening	Qualitative screening	Quantitative phosphorus mass concentration /mg·L^−1^	Qualitative screening	Quantification of IAA concentration /mg·L^−1^	Qualitative screening	Quantification of ACC deaminase activity /U·mg^−1^	Qualitative screening
L6	+	+	2.31	+	2.65	−	−	orange
LM3	+	+	2.16	+	5.32	+	3.75	light orange

**Table 3:** Analysis of resist stress ability of L6 and LM3.

MHB	Antagonism of pathogenic bacteria		Heavy metal antagonism (transparent circle/cm)		
	Antagonistic strains	Inhibition rate/%	50 mmol/L Cd^2+^	400 mmol/L Cu^2+^	100 mmol/L Zn^2+^
L6	*F. sporotrichioides*	31.25	2.7	1.37	1
	*B. dothidea*	38.6			
LM3	*F. sporotrichioides*	51.6	0.53	1.33	0.5

## Discussion

Numerous studies have confirmed that the presence of MHB promotes both mycorrhizal synthesis efficiency and plant growth (Miransari, 2011; Adeleke and Dames, 2014). At the same time, there is a synergistic evolutionary mechanism between mycorrhizal fungi and helper bacteria; MHB can interact with different fungal species, but with different effects. Some MHB strains can synergize with a wide range of mycorrhizal fungi of different species; for example, *Pseudomonad monteilii* HR13 can bind to several fungal isolates, and all of them can promote the colonization of ectomycorrhizal and tussock mycorrhizae of *Acacia* (Duponnois and Plenchette, 2003). In contrast, some MHB are extremely selective and can only interact with a particular fungus (Leyva-Rojas et al., 2020). Since MHB and mycorrhizal fungi have specificity for mutual selection during co-evolution, it is necessary to screen MHB for different mycorrhizal types.

The promotion of mycorrhizal fungi by MHB is the result of a combination of several aspects, including the fact that MHB can produce a variety of metabolites to promote the growth of mycorrhizal fungi, which is also one of the most important roles of MHB (Britton et al., 2021). Therefore, mycelial growth after mycorrhizal fungal-bacterial isolate interactions also becomes an important indicator for the rapid screening of MHB. A novel metabolite, Auxofuran, was isolated from the soluble metabolites of *Streptomyces* AcH505, which showed the best growth-promoting effect on *A. muscaria* at concentrations of 0.01–1 μmol/L (Deveau et al., 2007). Aspray et al. (2013) found that the *Paenibacillus* sp. EJP73 metabolite 2,5-di-isopropyl pyrazine promoted the root colonization of *Lactarius rufus*. In this study, the extracellular metabolites of L6 and LM3 were co-cultured with *O. maius* 143, and the extracellular metabolites of both potential MHB strains were found to promote the mycelial growth of *O. maius* 143, which increased by 40.9 and 57.1%, respectively, compared with the control. In addition, L6 and LM3 were tentatively identified as potential MHB strains.

MHB play an important role in mycorrhizal-plant interactions by increasing the rate of mycorrhizal fungal infestation of plant seedlings, enhancing their colonization status, and thus promoting plant growth. The synergistic effect of *P. fluorescens* SBW25 and *Laccaria bicolor* promoted the mycorrhizal colonization of poplar roots and inhibited the antifungal response of poplar roots, improving the ability of *L. bicolor* to form ectomycorrhizal interactions with poplar roots (Shinde et al., 2019). *B. cereus* HBl2 and HB59 were isolated and screened from the *Pinus thunbergii*-*Boletus edulis* rhizosphere and inoculated with *B. edulis* to promote the growth and mycorrhizal formation of *P. thunbergia* (Wu et al., 2012). A study of the double inoculation of *Glomus intraradices* and *Pseudomonas* spp. with *Acacia senegal* also showed that the biomass indices of plants with double inoculation were significantly higher than those with single inoculation, and inoculation with MHB significantly increased the mycorrhizal infection rate (Duponnois and Plenchette, 2003). The results of this study also showed that the co-inoculation of *O. maius* 143 with L6 or LM3 significantly increased the mycorrhizal infestation rate of the blueberry root system; improved the leaf GOGAT, GDH, GS, and NR enzyme activities; and effectively promoted the growth and nutrient uptake of blueberry. This suggests that one of the mechanisms of action of MHB strains L6 and LM3 is to promote the infestation of *O. maius* 143 in the root system of blueberry seedlings and improve the nutrient uptake of blueberries.

**Figure 6:**
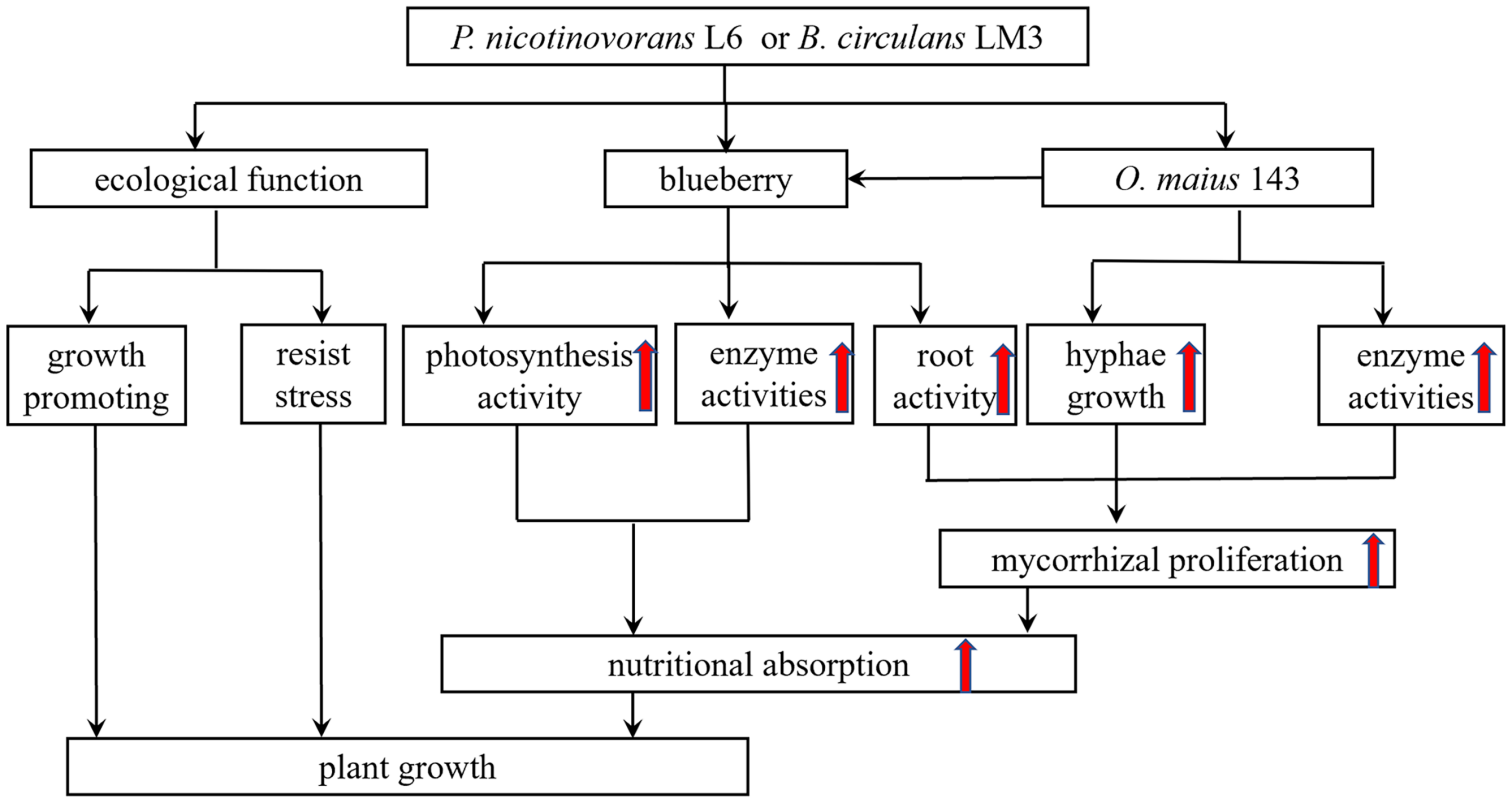
A proposed model illustrating the mechanism through which mycorrhizal helper bacterium *P. nicotinovorans* L6 or *B. circulans* LM3 improve the growth of blueberry.

The mycorrhizal formation is a process of mutual antagonism and eventual equilibrium. During the interaction between ErMF and plants, fungal spores germinate to form mycelia and produce cell wall degrading enzymes. There are many reports on the secretion of cell wall-degrading enzymes (such as chitinase, β-1,4-glucanase, β-glucosidase and polygalacturonase) by ErMF (Smith and David, 2008; Lin et al., 2011). These enzymes promote the decomposition of plant residues in the soil by mycorrhizal fungi and provide nutrients to the plants. In addition, these enzymes also play an important role in the invasion process of mycorrhizal fungi, which can disrupt plant cell walls and facilitate mycelial invasion to form symbioses. It has been shown that MHB enhance the colonization of plant roots by injecting molecules into fungal spores, by producing bacterial volatiles, and by increasing the activity of fungal cell wall degrading enzymes. Wang et al. (2022) found that *Hymenochaete* sp. Rl cultured in ZPD medium containing fermentation products of *B. pumilus* HR10 compared with no bacterial inoculation significantly increased mycelial β-1,3-glucanase and laccase activities. *Paenibacillus alvei* can promote mycorrhizal colonization by degrading cellulose in the environment to N-acetylglucosamine through a strong chitinase synthesis capacity. Some MHB metabolites were also found to contain cell wall degrading enzymes, so some MHB can promote mycorrhizal fungi to produce cell wall degrading enzymes, and the two act synergistically to destroy plant cell walls, thus promoting mycorrhizal fungi to colonize plant roots and mycorrhizal synthesis (Bourles et al., 2020). In this study, we co-cultured L6 and LM3 extracellular metabolites with *O. maius* 143 and found that co-inoculation significantly increased the cell wall-degrading enzyme activity of *O. maius* 143. We speculate that some MHB can exhibit mycorrhizal auxotrophy by increasing the cell wall degrading enzyme activity of mycorrhizal fungi, disrupting plant cell wall components, and facilitating mycelial invasion to form symbioses, thus enhancing their colonization status in plant roots (Figure 6).

The MHB that have been isolated and identified include G^+^ bacteria of the genera *Streptomyces*, *Bacillus*, *Rhodococcus*, and *Paenibacillus*, and G^−^ bacteria of the genera *Burkholderia*, *Pseudomonas*, and *Agrobacterium*. In this study, two strains of MHB were isolated and screened from the inter-rhizosphere soil of *V. uliginosum*, and L6 was initially identified as *P. nicotinovorans* and LM3 as *B. circulans* by morphological, physiological and biochemical as well as 16S rDNA sequence analyses. It is worth mentioning that in this study, the treatments of L6 and LM3 alone also had significant growth-promoting effects on blueberry growth, and all growth indices were better than those in the control treatment, indicating that L6 and LM3 also have other important growth-promoting mechanisms and good potential for application.

Later, by investigating the growth-promoting and inhibition functions of L6 and LM3, it was found that both strains could fix nitrogen, solubilize, produce IAA and produce IAA and iron carriers, which is consistent with the results in the study conducted by Luo et al. (2021) who found that the inter-root probiotic bacteria of *Pinus sylvestris* var. *mongolica* could fix nitrogen, solubilize and produce iron carriers, as well as exhibit a high IAA production capacity. The phytohormone IAA also acts as a signal for biofilm formation to support the bacterial colonization of plant roots (Ansari and Ahmad, 2019). The production of plant growth-promoting agents by bacteria, including IAA and their regulators, such as ACC deaminase, can also contribute to promoting plant growth. In addition, LM3 produced ACC deaminase with an enzyme activity of 3.75 U/mg, while L6 did not produce ACC deaminase. In anti-pathogenic assays, both L6 and LM3 inhibited the growth of phytopathogenic fungi and showed significant antagonistic effects against the heavy metals Cd^2+^, Cu^2+^, and Zn^2+^. MHB can improve the physical and chemical properties of inter-root soil by fixing nitrogen, phosphorus, and potassium, and can promote plant growth and development by secreting phytohormones such as IAA, gibberellin and growth hormone. MHB can secrete iron carriers to chelate Fe(OH)_3_ that cannot be used in the soil, forming Fe-iron carrier chelates to assist plants to obtain and use iron from the soil environment, thus competing with phytopathogenic bacteria for iron to inhibit the growth of pathogenic bacteria; improving the defense ability of plants by antagonizing pathogenic bacteria and heavy metals; and exhibiting ACC deaminase activity to hydrolyze ACC into α-butanone. LM3 also has ACC deaminase activity, which hydrolyzes ACC into α-butyric acid to reduce the content of ethylene inside the plant and improve plant stress resistance. Therefore, MHB are important in promoting the colonization of mycorrhizal fungi in plant roots and promoting plant growth (Figure 6).

## Conclusion

In this study, two strains of MHB, *P. nicotinovorans* L6 and *B. circulans* LM3, were screened from the roots of *V. uliginosum*. The co-inoculation of *P. nicotinovorans* L6 or *B. circulans* LM3 with *O. maius* 143 promoted blueberry seedling growth and increased the fungal colonization rate and root activity. Root growth and increased root activity contributed to nutrient uptake and increased GOGAT, GDH, GS, and NR enzyme activities in the blueberry leaves. Increased photosynthesis also contributed to the growth of the blueberries. The interactions between MHB and mycorrhizal fungi are beneficial to the growth of blueberry seedlings. This study provides a theoretical reference for the development of microbial agents and further research on the mechanism of MHB-mycorrhizal fungi-host plant interactions.

## Data availability statement

The datasets presented in this study can be found in online repositories. The names of the repository/repositories and accession number(s) can be found in the article/Supplementary material.

## Author contributions

ZY, HD, and SZ performed the experiments and analyzed the data. ZY, JJ, and HZ analyzed these data. ZY, HY, and LL wrote the manuscript. HY and LL designed the experiments and provided lab space and funding for this work. All authors read and approved the manuscript.

## Funding

The work was supported by the National Natural Science Foundation of China (31971694 and 32071806), the Fundamental Research Funds for the Central Universities (No. 2572021AW18), and the Natural Science Foundation of Heilongjiang Province (LH2020C101).

## Conflict of interest

The authors declare that the research was conducted in the absence of any commercial or financial relationships that could be construed as a potential conflict of interest.

## Publisher’s note

All claims expressed in this article are solely those of the authors and do not necessarily represent those of their affiliated organizations, or those of the publisher, the editors and the reviewers. Any product that may be evaluated in this article, or claim that may be made by its manufacturer, is not guaranteed or endorsed by the publisher.
