# Supplementary material for: Isolation and identification of mycorrhizal helper bacteria of Vaccinium uliginosum and their interaction with mycorrhizal fungi
Source: Front Microbiol. 2023 Apr 18;14:1180319. doi: 10.3389/fmicb.2023.1180319 (PMC10151510; doi:10.3389/fmicb.2023.1180319)
Supplement: Supplementary file 1 [file Table_1.DOCX]

**Table S1.** Primer sequences used in this study

| Gene name | Protein name | Function | Forward primer sequences | Reverse primer sequences |
| --- | --- | --- | --- | --- |
| *GH45* | β-1, 4 glucanase | glycoside hydrolase | CCGTAATGGTCTGGATG | GGTAAAACTGAGGGGTG |
| *GH1* | β- glucosidase | glycoside hydrolase | TACGCTTGCTGGCTA | CTCGTGCTCTTTCCG |
| *GH28* | Polygalacturonase | Pectin depolymerase | GGTCTGGAGTCTTTC | TAACTTCAGTGGCAT |
| *GH18* | Chitinase | glycoside hydrolase | ATGATGCTGATGGCGT | TATTTGCTTCGGGGAG |
